# From individualised treatment goals to personalised rehabilitation in osteoarthritis: a longitudinal prospective mapping study using the WHO international classification for functioning, disability and health

**DOI:** 10.1080/07853890.2022.2131326

**Published:** 2022-10-19

**Authors:** Sinisa Stefanac, Claudia Oppenauer, Michael Zauner, Martina Durechova, Daffodil Dioso, Daniel Aletaha, Gerhard Hobusch, Reinhard Windhager, Tanja Stamm

**Affiliations:** a Institute for Outcomes Research, Centre for Medical Statistics, Informatics and Intelligent Systems, Medical University of Vienna, Vienna, Austria; b Ludwig Boltzmann Institute for Arthritis and Rehabilitation, Vienna, Austria; c Karl Landsteiner Private University for Health Sciences, Krems, Lower Austria; d Clinical Department of Rheumatology, Medical University of Vienna, Vienna, Austria; e Department of Orthopaedics, Medical University of Vienna, Vienna, Austria

**Keywords:** Goal attainment scale, individualised treatment goals, ICF, osteoarthritis, Austria

## Abstract

**Background/Objective(s)/Introduction:**

In clinical practice, treatment goals are often set up without exploring what patients really want. We, therefore, collected individualised treatment goals of patients with osteoarthritis (OA), categorised and mapped them to the World Health Organisation International Classification for Functioning, Disability and Health (ICF).

**Patients/Materials and Methods:**

A longitudinal prospective cohort study was conducted (2019–2021). We used descriptive statistics and Chi^2^/Fisher’s Exact Tests, where appropriate, as well as Kruskal-Wallis-Tests for the mean score ranks of the patients’ goals.

**Results:**

In total, 305 goals reported by 132 participants were analysed (267 women vs. 38 men). The top 3 ICF categories were sensation of pain (ICF:b280), mobility of joint (ICF:b710) and muscle power functions (ICF:b730). Overall, 51% of all individually reported functional goals were achieved after 3 months. Men were more likely to achieve their goals than women (*p* = 0.009). The majority of the “*very important*” goals (51%) and “*very difficult*” goals (57%) was not improved. Goals’ mean score ranks significantly differed between baseline and follow-up.

**Conclusion(s):**

As the human lifespan as well as the number of people affected by OA worldwide increase, there is a growing need to identify and evaluate rehabilitation outcomes that are relevant to people with OA.

**Key Messages**
- Treat-to-target agreements between patients and health care providers present a step towards more personalised precision medicine, which will eventually lead to better reported functional and health outcomes.
- In patients with osteoarthritis, the Goal Attainment Scale instrument can be used to measure health outcomes at different time points and its content may be linked to ICF providing a unified language and conceptual scientific basis.

## Introduction

According to the World Health Organisation (WHO), rheumatic and musculoskeletal diseases (RMDs) are one of the top five leading causes of disability worldwide [1,2]. They significantly limit mobility and dexterity, affect a person’s quality of life (QoL) and lead to early retirement as well as reduced possibilities to participate in the activities of daily living (ADLs) [3–7]. Osteoarthritis (OA) is the most common RMD with a high prevalence in older age and is also a major cause of disability among older individuals [8,9]. Osteoarthritis is a slowly progressing degenerative joint disease, characterised by the deterioration of cartilage in joints, changes in subchondral bone as well as in other joint tissues such as the ligaments, which results in bones rubbing together leading to stiffness, pain and impaired movement [10–21].

Functioning and independence in daily life are essential for health and wellbeing of all people [22,23]. A loss of functioning and independence because of a health condition or comorbidity is described as activity limitation and/or participation restriction. Both can be related to the individual (e.g. loss of a specific body function), to the environment (e.g. a lack of support, a lack of accessibility and other) and/or to the context (e.g. cultural, personal and other) [24]. The International Classification for Functioning, Disability, and Health (ICF) is the WHO framework for measuring functioning. In the ICF, functioning is described as an interaction between body structures and body functions, activities, and participation, as well as personalised and environmental factors [24]. If people with OA experience activity limitations and/or participation restrictions, outcome measurements need to take these aspects into account.

Disease management requires precise and accurate assessment tools, and due to the need to justify interventions and prove their effectiveness, the pressure to use standardised outcome sets is increasing [25]. For clinical decision-making, changing policies and funding, patient-reported outcomes play a crucial role, as they ensure that the perspective of each patient is taken into consideration [26,27]. These outcome measures, especially in chronic diseases may provide valuable information about patients’ experience, satisfaction, and perceived health status, but they could also demonstrate the effectiveness of different interventions, such as pharmacological as well as non-pharmacological treatment, and show areas where more tailored approaches are required. However, to ensure that the used instruments accurately assess what they are supposed to measure, they must demonstrate evidence of psychometric properties including reliability and validity and they should be used in a standardised way [27–29].

An example for such an assessment tool is the Goal Attainment Scale (GAS). The GAS asks patients to self-define treatment goals and intervention priorities in a standardised way supported by health professionals [30]. A substantial body of evidence shows that patients are more motivated to change their behaviour and improve rehabilitation outcomes if treatment goals are clearly defined and relevant to them [31–35]. As several studies have established appropriate sensitivity, validity and reliability of GAS [30,36,37] it appears to be a sound measure for use in rehabilitation settings in people of all age [34,38]. Instead of setting up “a priori” outcomes, by using the GAS instrument in everyday practice, more individually important outcome targets may be captured [39]. Other potential benefits of the GAS include realistic patient expectations, a mutual understanding of the preferences about goal setting between patients and healthcare providers, improved clarity of therapy objectives, increased motivation due to seeing one’s personal improvement and increased patient satisfaction [38,40].

The GAS instrument is widely used to identify and evaluate relevant goals of an individual. To the best of our knowledge, several studies have already linked the GAS goals to the ICF in paediatrics including different diagnostic aims such as children and adolescents with cerebral palsy, Down syndrome, spina bifida, spinal cord injury as well as children with gastrostomy tube placement [41,42].

Moreover, other studies linked the GAS goals to adults with neurological and psychological conditions such as stroke, spasticity, multiple sclerosis as well as dystonic type of cerebral palsy [43–45], however linking of the GAS goals in adults with OA to the ICF components has not yet been done [46]. This is of importance, as OA is different from these congenital and acquired developmental, neurological, and psychological conditions since it affects primarily functioning of the musculoskeletal system and leads to pain, stiffness of joints, decline in physical functioning and reduced QoL.

Thus, the aim of this study was to categorise individually reported functional goals of patients with OA and link them to the ICF according to the established standard linking rules [47–49]. Linking the GAS to the ICF will provide an overview of individual goals in people with OA, underlining the current functional challenges, but equally important, it will facilitate future professional communication between patients and health-care service providers and thus improve forthcoming health-care planning and rehabilitation value.

## Patients/materials and methods

### Design

This longitudinal prospective cohort study was conducted at the Clinical Department of Rheumatology and the University Clinic for Orthopaedic and Trauma Surgery at the Medical University of Vienna (MUW) from September 2019 until September 2021.

Before March 2020, the GAS questionnaire was administered in a face-to-face setting with the participants at the rheumatology and orthopaedic outpatient clinics at the MUW. Patients who visited the outpatient clinics, were screened by us for inclusion in the study and baseline as well as follow up assessments were performed.

However, due to the COVID-19 pandemic, regulations from the authorities and hospital restrictions, the face-to-face recruitment was replaced with telephone calls. During the phone call, the study information and aims were presented. If patients agreed to participate in this study, immediately after the phone call, envelopes containing the informed consent, study information and one-way test equipment were sent to participants’ homes. On an agreed date and time (which was usually two weeks after the initial phone call), inclusion in the study commenced. Afterwards, patients returned the envelopes with their signed informed consent and filled-in questionnaires to the MUW. After a baseline assessment was done, all patients were asked to participate one more time in another telephone follow-up call which occurred 3 months after the baseline date. In terms of questionnaire administration and answers provided, no differences were observed between face-to-face recruitment and telephone calls.

Since the aim of this paper is to cluster individually reported functional goals of patients with OA and link them to the ICF, no intervention was given. The original protocol as well as all amendments were approved by the Ethical Committee of the MUW.

### Participants

People diagnosed with OA according to the ACR/EULAR criteria [50,51] from the “Better Life in Osteoarthritis Registry (BLOAR)” [52] were recruited at their first visit if they were ≥18 years, and have provided oral and written informed consent according to the Declaration of Helsinki [53]. The exclusion criteria were the presence of any neuro-psycho-motor disease.

### Goal attainment scale

The GAS questionnaire is a standardised instrument that captures individuals’ goals and measures the extent to which they are achieved, e.g. after rehabilitation or other interventions [30,38,54]. Each participant sets up their own goals, following the “SMART” criteria which request goals to be Specific, Measurable, Acceptable, and defined in Time; this allows monitoring the patient’s progress in a reliable manner [30,38,55]. Every participant ranks the importance of each goal on a 4-point scale from 0–3, where (0) = not at all important, (1) = little important, (2) = moderately important, and (3) = very important. The difficulty of each goal is also ranked on a similar scale from 0–3, where (0) = not at all difficult, (1) = little difficult, (2) = moderately difficult, and (3) = very difficult. Guided by a health professional (SS), all participants identified their individual goals and levels of their current and expected performance. The measurement of goal achievement was taken on a 5-point scale from −2 to +2 according to the GAS guidelines [40,54], where (-2) = much less than expected, (-1) = a little bit less than expected, (0) = as expected, (+1) = a little bit more than expected and (+2) = much more than expected. For example, if the patient had problems with walking short distances, all expected outcomes (“levels”) were defined by patient and health professional together on a 5-point scale. For instance, (0) = the patient will walk to the park 300 m without stopping or taking a rest, (+1) the patient will walk to the park 450 m without stopping or taking a rest, (+2) = the patient will walk to the park and return home without stopping or taking a rest (600 m), (−1) = the patient will walk only half way to the park and needs to stop or take a rest and (−2) = the patient cannot leave the house.

All goals were weighted by the participants’ importance and difficulty using a 4-point scale, where 0 = not at all important/difficult, 1 = of little importance/difficulty, 2 = moderately important/difficult and 3 = very important/difficult. The scores were later transformed into a numerical T-score by using the GAS-formula according to the GAS rules and standards [40,54].

### *Procedure*s

Before linking the goals to the ICF categories, authors who were in charge for the mapping (SS, CO, and TS) acquired a good knowledge of the conceptual and taxonomical fundaments of the ICF as well as the established standard ICF linking rules [47–49]. SS is an occupational therapist, currently working as global market access manager in the medical technology industry and has 5 years of experience in the ICF linking. CO is clinical psychologist who has 10 years of experience in the ICF linking. TS is a professor for Outcomes Research at the Medical University of Vienna. She has a background in health science, educational science, business administration, human biology and occupational therapy. Since more than 20 years, TS conducted ICF-based research including instruments’ item linking, especially in rheumatology, rehabilitation and other chronic conditions.

To ensure accuracy, grouping and linking was done according to the accepted protocol which have been developed to link health measures in a specific and precise manner [47–49]. First, we explored all goals reported by one individual. All goals that shared the same meaning were grouped. This was done across participants in a consistent manner. Afterwards, all individually reported functional goals (subcategories in the ICF classification system) were linked to the most precise ICF categories that addressed individuals’ specific context. For example, subcategory “*pain in the knee*” (ICF code: b28015 – Pain in lower limb) and “*pain in the shoulder*” (ICF code: b28014 – Pain in upper limb) were grouped together in the most precise ICF category “*pain*” (ICF code: b280 – Sensation of pain), while for instance, “*walking 15 meters*” (ICF code: d4500 – Walking short distances) and “*walking for one hour*” (ICF code: d4501 – Walking long distances) were grouped as the ICF category “*walking*” (d450 – Walking). To avoid coding mistakes, all goals were initially coded by one researcher (SS), and double checked by another trained independent researcher (CO). Any discrepancies were solved by the third researcher with extensive experience in ICF linking (TS). All researchers were trained in the ICF linking rules.

### Statistical analysis

The sociodemographic characteristics of participants were presented with descriptive statistics. Metric variables (age and year of diagnosis) which were not normally distributed were described with median, interquartile range, minimum, and maximum scores. Histograms and Kolmogorov-Smirnov tests were used to assess data distribution. In case of non-normal distributions, non-parametric methods were selected.

The main ICF categories with the corresponding GAS T-scores were analysed according to the guidelines [40,54]. The distribution of the GAS summary scores was described with univariate descriptive statistics and graphics. The Chi^2^–tests for independence or alternatively, Fisher’s Exact Tests if group sample sizes were below five, were used to determine differences in frequency of categorical variables (sex, goal achievement, expected outcome, goal importance and goal difficulty). The Bonferroni-Holm correction was utilised as a method for multiple testing adjustment [56–58]. The data extraction was done using Microsoft Office Excel program, and the statical analyses were performed using the IBM^®^ SPSS^®^ version 27.

## Results

In total, 132 participants were recruited with a median age of 63.9 (IQR:16). Women were represented at a higher rate (88%; *n* = 111; 95% CI: 0.87–0.91) as compared to men. This is in line with the gender distribution in OA [13,59–65]. Since each participant could specify up to three personalised GAS functional priorities, at the end of the study we have analysed 305 individually reported functional goals (267 from women vs. 38 from men).

In total, we have linked 13 ICF categories and presented 2 additional individually reported group of goals that were not defined in the ICF coding system (“*unchanged/maintain current functional status*” and “*making less breaks*”). The category “*unchanged/maintain current functional status*” means that the participants were satisfied with their current functional status when they entered the study, and they wanted to maintain that same level of functionality until the follow-up. The second category was “*making less breaks*”, which was communicated when the participants wanted to reduce the frequency of doing a total number of breaks while performing certain ADLs.

Most of the reported functional goals with 66.2% (*n* = 202) were linked to the ICF “*b-Body function*” component while 5.2% (*n* = 16) were associated with ICF “*d-Activities and participation*” component. Other 28.6% (*n* = 87) were related to the two additional categories that are not ICF coded (“*unchanged/maintain current functional status*” and “*making less breaks*”). Sociodemographic characteristics of the study population stratified by those who improved and did not improve their functional goals is presented in Table 1.

**Table 1.** Sociodemographic characteristics of the study population (*N* = 132) stratified by those who improved and did not improve their individually reported functional goals.

Variables	Total goals	Goals achieved	Goals not achieved	*p* Value
	*N* = 305	*n* = 154	*n* = 151	
Age				0.016
Range; median years (IQR)	35–91; 63.9 (15)	35–91; 64.3 (15)	45–86; 62.3 (18)	
				0.037
≤64 %(*n*)	56.7 (173)	45.1 (78)	54.9 (95)	
≥65 %(*n*)	43.3 (132)	57.6 (76)	42.4 (56)	
Sex				0.009
Women %(*n*)	87.5 (267)	47.6 (127)	52.4 (140)	
Men %(*n*)	12.5 (38)	71.1 (27)	28.9 (11)	
Years of living with OA diagnosis				0.001
5 or less years %(*n*)	22 (67)	32.8 (22)	67.2 (45)	
More than 5 years %(*n*)	78 (238)	55.5 (132)	44.5 (106)	
ICF components				<0.001
b-Body function %(*n*)	66.2 (202)	35.6 (72)	64.4 (130)	
d–Activities and participation %(*n*)	5.2 (16)	56.3 (9)	43.8 (7)	
Other^a^ %(*n*)	28.5 (87)	83.9 (73)	16.1 (14)	
Importance				0.050
Not at all important %(*n*)	0 (0)	0 (0)	0 (0)	
Of little importance %(*n*)	0 (0)	0 (0)	0 (0)	
Moderately important %(*n*)	9.5 (29)	69 (20)	31 (9)	
Very important %(*n*)	90.5 (276)	48.6 (134)	51.4 (142)	
Difficulty				<0.001
Not at all difficult %(*n*)	5.9 (18)	100 (18)	0 (0)	
Of little difficulty %(*n*)	4.9 (15)	66.7 (10)	33.3 (5)	
Moderately difficult %(*n*)	38 (116)	51.7 (60)	48.3 (56)	
Very difficult %(*n*)	51.1 (156)	42.3 (66)	57.7 (90)	

^a^Contain additional goals that were not ICF coded (1. “*Unchanged/maintain current functional status*” 2. “*Making less breaks*”).

### Distribution of individually reported functional goals across and within gender

Overall, the top three most reported ICF categories where “*reduction of pain*” (ICF category: b280 - “*sensation of pain*”) with 43.6% (*n* = 133), followed by “*improving joint mobility*” (ICF: b710 - “*mobility of joint functions*) with 10.2% (*n* = 31) and “*increasing muscle power*” (ICF: b730 - “*muscle power functions*”) with 9.5% (*n* = 29).

**Figure 1.**
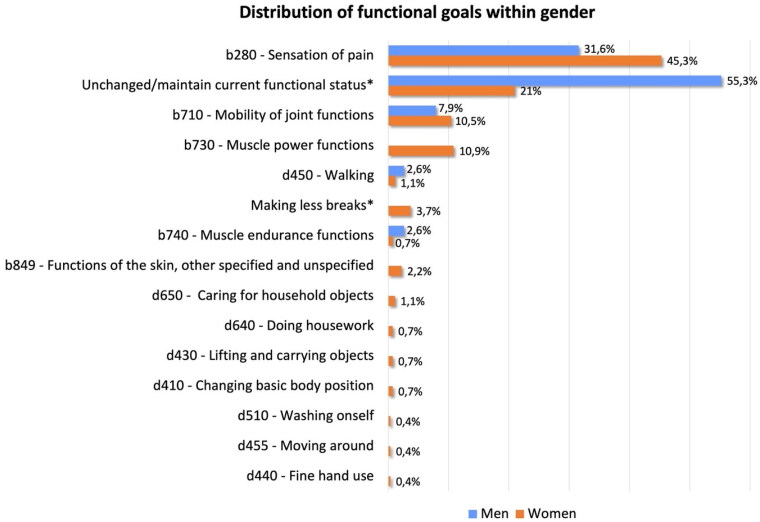
Distribution of the individually reported functional goals within gender.

Statistical significances were found between individually reported functional goals and gender (*p* = 0.015). “*Reduction of pain*” was the most reported functional goal in women with OA (45.3%; *n* = 121), followed by the “*unchanged/maintaining current functional status*” with 21% (*n* = 56) and “*muscle power functions*” with 10.9% (*n* = 29). Interestingly, in men, the most frequently reported functional goal with 55.3% (*n* = 21) was “*unchanged/maintaining current functional status*” followed by the “*reduction of pain*” with 31.6% (*n* = 12) and “improving *joint mobility*” category (7.9%; *n* = 3). Distribution of the individually reported functional goals within gender is shown in Figure 1.

### Goal improvement based on the duration of OA diagnosis, importance and difficulty levels

Out of all reported functional goals, just a little bit more than half (50.5%; *n* = 154) were improved at the time of follow-up. As shown in Table 1, men improved more functional goals than women (71.1% vs. 47.6%; *p* = 0.009), and participants who were diagnosed with OA for more than five years improved most of their goals (55.5%; *n* = 132), in contrast to 32.8% (*n* = 22) of those participants who were living with OA for 5 or less years (*p* = 0.001). At the time of follow-up, 71.1% (*n* = 27) of all functional goals reported by men were improved, as compared to 47.6% (*n* = 127) reported by women.

In total, 69% (*n* = 20) of “*moderately important*” and 49% (*n* = 134) of “*very important*” goals were improved after a 3-month follow-up (*p* < 0.001). Most of the women (65.4%; *n* = 17) who marked their individual goals as “*moderately important*” enhanced their expected outcome after a follow-up period, while the greatest number of “*very important*” goals remained unimproved (54.4%; *n* = 131). On the other hand, all “*moderately important*” functional goals reported by men were accomplished within 3 months as well as 68.6% (*n* = 24) of “*very important*” goals.

As shown in Table 1, 57.7% (*n* = 90) of all individual goals that were marked as “*very difficult*” were not realised at the time of follow-up (*p* < 0.001). The majority of those goals were not improved in a group of participants who were diagnosed with OA in the last five years (68.8%; *n* = 22) as well as in group of those participants living with OA for more than five years (54.8%; *n* = 68). Frequency of the three most common and most difficult functional goals stratified by improved and not improved, is presented in Table 2.

**Table 2.** Frequency of the improved and not improved functional goals in patients with osteoarthritis.

	Total number of goals	Goals achieved	Goals not achieved
Overall goal improvement after 3 months follow-up	305	50.5% (*n* = 154)	49.5% (*n* = 151)
The 3 most frequent ICF goals			
Reduce of pain (ICF Category: b280)	133	40.6% (*n* = 54)	59.4% (*n* = 79)
Improve joint mobility (b710)	31	38.7% (*n* = 12)	61.3% (*n* = 19)
Increase muscle power (b730)	29	17.2% (*n* = 5)	82.8% (*n* = 24)
The 3 most difficult ICF goals			
Reduce of pain (ICF Category: b280)	82	42.7% (*n* = 35)	57.3% (*n* = 47)
Improve joint mobility (b710)	20	50% (*n* = 10)	50% (*n* = 10)
Increase muscle power (b730)	18	16.7% (*n* = 3)	83.3% (*n* = 15)
The 3 least likely ICF goals to be achieved			
Increase muscle power (ICF Category: b730)	29	17.2% (*n* = 5)	82.8% (*n* = 24)
Reduce swelling (b849)	6	0	100% (*n* = 6)
Caring for household objects (d650)	3	0	100% (*n* = 3)

**Figure 2.**
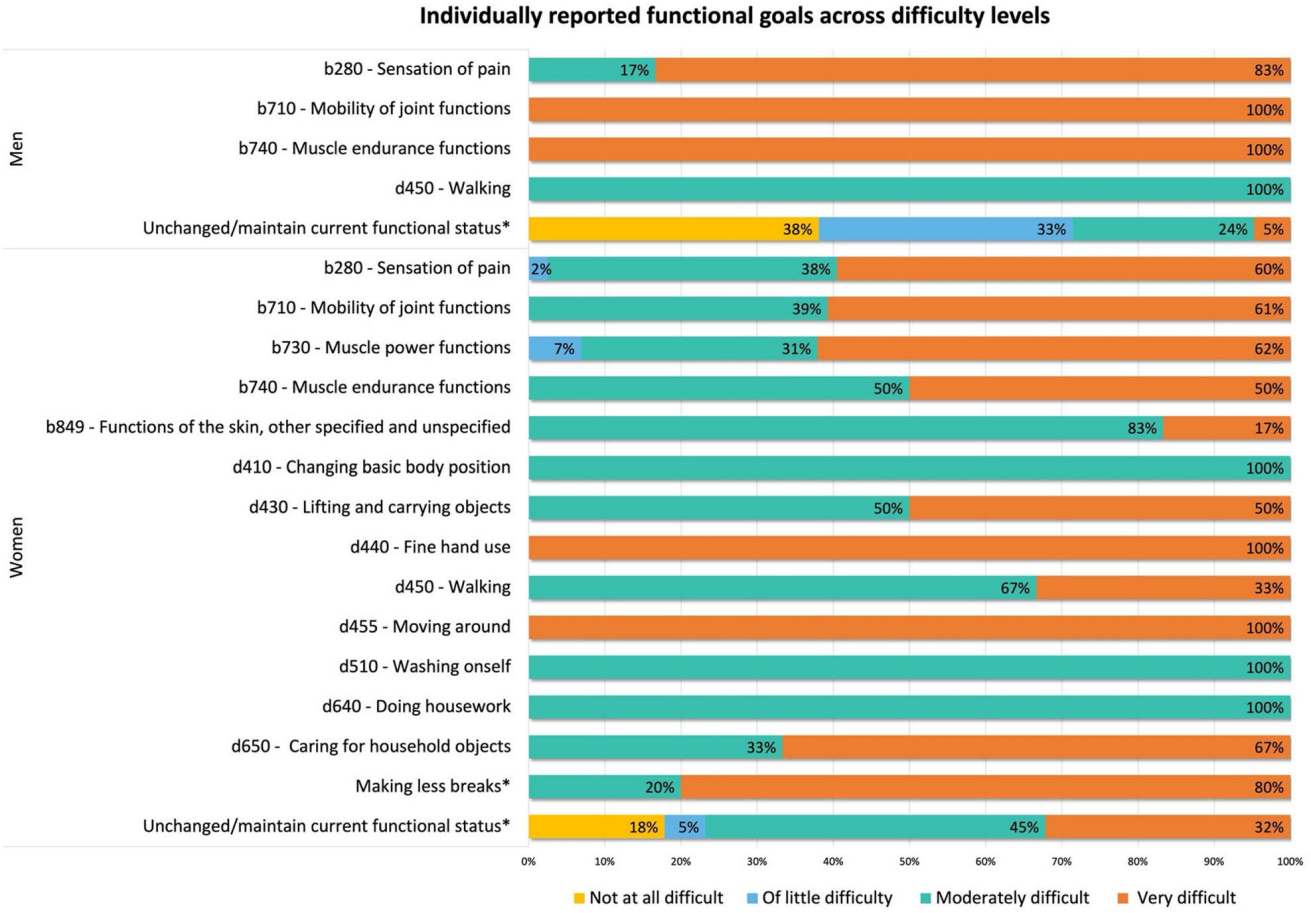
Distribution of the difficulty levels across individually reported functional goals.

Figure 2 shows distribution of the reported functional goals across difficulty levels and within gender. Overall, in the ICF component “*b-Body function*”, the majority of the reported functional goals were not improved (64.4%; *n* = 130), while most of the goals in the ICF component “*d–Activities and participation*” were realised (56.3%; *n* = 9; *p* < 0.001; Table 1).

### GAS-score ratings at baseline and follow-up

Kruskal-Wallis-Test showed a statistically significant difference in the overall GAS score between the individually reported difficulty levels across baseline (T1) and follow-up (T2). Table 3 shows the differences in the median GAS scores at T1 and T2.

**Table 3.** Differences of the GAS median scores between the individually reported difficulty levels at baseline T1 and follow-up T2.

Difficulty	GAS T1 score	GAS T2 score
	Median (IQR)	Median (IQR)
Not at all difficult	0 (0)	0 (0)
Of little difficulty	50.33 (0.17)	105 (370)
Moderately difficult	50.17 (0)	68.33 (166.67)
Very difficult	50.11 (0)	74.44 (111.11)

At T1, the Mann-Whitney-U-Test showed significant differences between “*not at all difficult*” and all other difficulty categories, while at T2 significant differences were found between “*of little difficulty*” and “*moderately difficult”* category.

## Discussion

This is the first study in osteoarthritis that aimed to cluster individually reported functional goals of patients in a functioning ICF framework using the GAS instrument, as well as to analyse their goal improvement after the 3-months follow-up period. In our study we had an overrepresentation of women, and people who were older than 60 years old, however that was expected and reported in previous studies [59–65].

### ICF categorisation

Two main ICF components that emerged from our 305 individually reported functional goals were “*b-Body functions*” and “*d-Activities and participation*”, while the remaining two ICF components, specifically “*e-Environmental factors*” or “*s -Body structures*” could not have been linked to our study participants. Linking the individually reported functional goals to the ICF components has been differently reported among existing literature. Previous studies done in people with OA by using other than the GAS measuring instrument, have already associated their functional goals to some or all four ICF components [46,66]. In one study done by Weigl *et al.* [66], authors managed to link the items of the Western Ontario and McMaster Universities Arthritis Index (WOMAC) and the Lequesne-Algofunctional questionnaire to 29 ICF categories, out of which 5 belong to the ICF “*b-Body functions*”, 23 to the ICF “*d-Activities and participation*” and 1 to the ICF “*e-Environmental factors*” component. The WOMAC questionnaire is frequently used in the evaluation of hip and knee OA [67], while the Lequesne-Algofunctional index [68] is widely used to evaluate functional abilities and the discomfort felt by patients suffering from knee OA.

A recent systematic review that was done by Povlak and Valdes [46], revealed 5 RCT studies that included patients with hand OA and RA. Examples of outcome measures that measured pain included the Michigan Hand Questionnaire, Numeric Pain Scale and the Visual Analog Scale, while for the assessment of QoL the EuroQol 5-Dimension Questionnaire together with the World Health Organise Quality of Life questionnaire was used. In order to address specific hand OA limitations across wide range of domains, the Canadian Occupational Performance Measure was used. The authors reported linking the measures to “*s -Body structures*” and “*b-Body functions*” in 50% (*n* = 6) and “*d-Activities and participation*” in 25% (*n* = 3). One measure (8.3%) was linked to “*e-Environmental factors*” whereas 2 (16.7%) outcome measures addressed QoL factors.

One of the reasons for different ICF-categories between our study and these done by Weigl *et al.* [66] and Povlak and Valdes [46], may be the use of different questionnaires such as the WOMAC, Lequesne-Algofunctional questionnaire, COPM, the EuroQol 5-Dimension Questionnaire together with the World Health Organise Quality of Life questionnaire. It is important to emphasise that to the best of authors’ knowledge, linking of GAS goals in adults with OA to the ICF components has not yet been done, which can also be confirmed in the latest systematic review in patients with OA [46].

Considering a very detailed ICF classification system when describing functional impairments related to participants’ ADLs [66], we were expecting that most of the individually reported functional goals would be associated with the “*activities and participation*” component. Interestingly, in our study, the activities and participation component are underrepresented with only 5.2%, shifting the focus from the important and problematic ADLs such as self-care, mobility, and productivity onto the body functions, in particular sensation of pain, muscle power and muscle endurance as well as joint mobility and functions of the skin.

In view of the fact that OA is a debilitating and chronic disease, mapping the functional outcome measures in people with OA to the ICF categories and components, might provide a broader understanding of the existing challenges in people with OA, and help the (non)medical professionals to provide more tailored approach that might lead to greater functional outcomes [46].

### Goal attainment across the ICF classification system

Our results showed that just a little bit over the half of all individually reported functional goals were improved after a 3-months follow-up period. This was the most surprizing finding, however, the fact that most of this study was done during the COVID-19 pandemic and multiple lockdowns, where participants did not have a frequent access to physicians and other health professionals as well as different therapeutic programs, should be taken into consideration. Some of the negative consequences of the COVID-19 pandemic on our study included a steep decline in the total number of patients who attended the outpatient rehabilitation sessions. For people with OA, this pandemic has partially or completely obstructed the provision of services such as doctor’s visits, pain management, occupational therapy, physiotherapy, dietician services, educational and other interventions which are an essential component in rehabilitation [69]. In order to keep promoting health, preventing functional decline and maintaining the best possible outcome, post-pandemic care needs to be reorganised and medical services must be pandemic ready in the future.

As stated in the results section, “*sensation of pain*” was the most reported functional goal, particularly in women, but that is not surprising as the several studies suggest that women with OA experience more pain than men [69,70]. Despite the COVID-19 situation in Austria, we have assumed that at least the reduction of pain would have been improved in the time frame of 3 months after the baseline. Interestingly, the pain reduction was not accomplished in most of the reported cases, and it got worse than it was initially disclosed at the beginning of this study. We did not expect that, however due to multiple lockdowns, access to health care professionals and pain management was limited.

Moreover, the second most frequently described functional goal, which was prevailing more in men than women, was “*unchanged/maintain current functional status*”. Even though this goal is not defined as a category in the ICF classification system, we have included it in our analysis as an additional group as it mattered to our study participants. Participants were setting up this goal when they did not have any OA health or activity related problems in everyday life, and they wanted to retain their current functional/health status. The majority of such reported goals were preserved until the end of follow-up, supporting the fact that the participant’s OA disease activity level has been subjectively sustained as it was initially described at baseline. Since the release of today’s ICF, a number of scientific articles, critiques as well as conceptual development and recognition in interdisciplinary team, contributed to constant ICF revisions and new versions [71]. For those reasons, all authors agreed to leave this goal and present it in the manuscript since it was very important to our research participants. Since ICF revisions happen every few years, it is our wish that over time more studies report on already established as well as other not ICF-indicated important functional and health goals, which together with the scientifical evidence might lead to further expansion of the ICF classification system in the future.

The fact that just a little bit under the half of all individually reported functional goals in our study remained unaccomplished, were without a doubt the most unexpected finding. The reasons for not improving those personal goals cannot be clearly interpreted from this dataset analysis. Nevertheless, they are most likely multifactorial and related to the fact that OA is a condition which does not easily improve, as well as to the national regulations and hospital restrictions which were implemented to limit the spread of COVID-19 infection and prevent unnecessary visits for patients and older adults with chronic diseases. COVID was a confounding variable in our study, and it has inevitably affected the rehabilitation service provision and goal achievement in the majority of our study participants. Further research is needed in patients with OA in order to draw conclusions about the effects of the COVID-19 pandemic on GAS questionnaire, goal achievement as well as the categorisation of the GAS goals within the ICF, and built upon our findings.

### Implications of our study outcome for rehabilitation and public health

Even though we did not have an intervention in our study as the aim of this paper was to cluster individually reported functional goals and link them to the ICF, defining precise and important goals is a crucial part of every treatment planning. In the clinical treatment and rehabilitation by using the GAS questionnaire, patient’s priorities can be captured, its progress can be quantified in a standardised way and thus better communicated to patients, their families and other organisations, including governmental institutions. On a broader public health level, for the first time we gathered information on the most important individual ICF goals in patients with OA. The information from our study can be used to justify the need for a more tailored rehabilitation approach in individuals with OA such as in occupational therapy, but also to reflect on already established other rehabilitation services and interventions.

#### Strengths and limitations

Limitations in our study include potential patient-reported bias such as year of diagnosis, importance, and difficulty levels as well as subjective weighting of the GAS scores. Importance and difficulty levels were collected on a scale from 0 “not at all important/not at all difficult” to 3 “very important/very difficult”, while the GAS scores were weighted according to the guidelines by individually reported importance and difficulty levels.

One of the strengths is that these results are reported for the first time in people with OA in Austria by using the GAS instrument. With the GAS instrument, we have captured the meaningful and important functional goals of people with OA and linked them for the first time to the ICF categories and components. Since several studies report different results after associating the functional goals in people with OA to the ICF categories and components, we therefore recommend that further studies should be done in this area and build upon our findings.

## Conclusions

For the first time, we categorised the most important individually reported functional goals of patients with OA and linked them to the ICF categories according to the established standard linking rules. As the human lifespan as well as the number of people affected by OA worldwide increase, there is a growing need to identify and evaluate rehabilitation outcomes that are relevant to people with OA. Since many widely used outcome measures already exist in everyday (non)clinical practice, choosing the right assessment tools where patients define their own goals is considered to be client-centered. Such treat-to-target agreements between patients and health care providers present a step towards more personalised precision medicine, which will eventually lead to better reported functional and health outcomes. In OA patients, the GAS instrument can be used to measure health outcomes at different time points and its content may be linked to ICF providing a unified language and conceptual scientific basis.

## Data Availability

Data sharing – not applicable.
